# Is Modern Dentistry a Specially Developed Branch of General Medicine?

**Published:** 1914-09-15

**Authors:** Otto Zsigmondy


### IS MODERN DENTISTRY A SPECIALLY DEVELOPED BRANCH OF GENERAL MEDICINE?

BY OTTO ZSIGMONDY.

Dr. Walkhoff, in recommending the German dentists should endeavor to obtain the title "Doctor der Zahnheilkunde"—a title analogous to the American "Doctor of Dental Surgery"—in order that there should be a general recognition of the fundamental dissociation of medicine and dentistry, has given fresh impetus to the discussion of the relationship of these two occupations. To support his contention that dentistry should hold a position altogether independent oi medicine, Walkhoff uses arguments which, if they were true, would draw together all the dental practitioners in the world into a bond of sympathy with regard to the purely national question of a suitable title for German dentists. These arguments must not remain unanswered. Dr. Walkhoff asks whether dentistry is a branch of the art of healing in general, as taught in the universities and thence carried out into practice, and replies to his own question by asserting that it is indeed a healing art—the art *de restitutio ad integrum* of the teeth and their annexes; but that, nevertheless, modern dentistry cannot be said to base itself solely, or even for the most part upon the teachings of medicine.

Medicine, he declares, is dependent, for its results, upon the proper functioning of those organs dealt with by dental science. The most important activity of medicine is the preparation of the way for the restoration of the general functions of the body by the removal of obstacles impeding the course of natural processes of healing. With regard to the teeth *there are no such processes* and there is no teaching in modern medicine which can point the way to the restitution of normal function in the mouth as it does for other organs of the body. It follows, therefore, that diseases of the teeth call for a therapeusis differing essentially from that of general medicine. It consists very largely in the replacement of organs either in part or in their entirety, and this substitution must be done in such a manner that neither the teeth nor the other parts of the body are injured by it. In these deviations of physiology and therapeutics lies the fundamental difference between modern general medicine and dentistry.

Walkhoff insists on the high value he sets on medical science and the need of using its resources as they apply to dentistry, in the fullest possible way, but, he adds, "so long as the medicine of today—or of tomorrow—fails to preserve the teeth by the methods and principles it applies to the body in general, so long it cannot justly claim that dentistry proceeds from it for the benefit of mankind and is one of its special branches."

He claims that neither general practitioners nor specialists for single organs of the body, nor again technicians who who have busied themselves merely manually with the disease of the teeth are possessed of the fundamental knowledge necessary to the main pursuit of dentistry—the preservation of the natural teeth—and that this knowledge is to be found only with the "dentists," only moreover with those dentists who are in possession of the combined sciences which bear upon their craft. It is, he asserts, manifest to-day that dentistry is progressing, can progress only along the lines laid down by the researches and the discoveries of these men.

It is flying in the face of the facts of dental history and of contemporary dentistry to maintain, as do the "stomatologists," that dentistry is a specialized branch of medicine and can therefore (!) be carried on only (!) by doctors. The art of preserving the teeth is a special art growing from its own roots and taking in and making use of surrounding knowledge whenever that knowledge has thrown light on its own special problems. The foundations of modern dentistry were laid in the sixties by Arthur, and later by Taft upon physical, chemical and general knowledge and the application of technological facts.

Walkhoff's claim for entirely distinct dental therapeutics breaks down when we consider first the question of the close relationship of the teeth with the neighboring organs—which Walkhoff himself brings within the province of dentistry—and secondly, the question of pyorrhea alveolaris. The flimsiness of his appeal to history appears as soon as one gets to close quarters with the makers of modern dentistry.

Who were the men who first taught and practiced the insertion of lasting fillings? Fauchard, in the eighteenth century, speaks with regret of the need for extracting teeth and inserting fillings, some of which lasted, to his knowledge thirty years and more. But the net results of experiments in filling by the French method was very slender, as was remarked by my father, Dr. Adolf Zsigmondy, who studied it in Vienna under Prof. Carabelli.

The art of filling in any sense worthy of the name was first taught in Vienna by the English dentist Morphy.

But it is to America that we must look if we would trace modern dentistry to its source. Long before Morphy introduced modern dentistry into Vienna there were in America institutions for the study of this specialty. Largely owing to the exertions of Drs. Hayden and Harris a school of dentistry was founded in Baltimore in 1840—while some twenty years earlier than that L. Koecker, a German, as a result of many years in America, wrote the book wherein are set forth the principles which have led dentistry forward to its present status. The book was written in English and appeared first in London. It lays stress upon the fact that operative dental surgery was nowhere so widespread and so far advanced as in America, though the literature of the subject was, so far, very slender. The author of this classic styles himself "Doctor of Medicine and Surgery," and has much to say of the dependence of dentistry upon these healing arts of which it is a branch. The sense of this relationship was very obvious to Drs. Hayden and Harris, the dentists who were instrumental in starting the first dental society, the first dental journal and in founding the dental school under the aegis of the medical faculty of the Baltimore University. Students in the Baltimore College of Dental Surgery were taught that dentistry is based upon a careful study of the principles of general medicine.

If, moreover, we consider the early literature of modern dentistry as a whole, we shall find that the insistence on its fundamental principle, the need for the conservation of the natural teeth comes from men who were doctors—there is no kind of support to be found for the suggestion that there were "dentists" who taught and practiced the truths of the dependence of their craft for its successful development, upon the sciences grouped together under the term general medicine. The "dentists" of the period had other things to do; they wandered from market to market That "modern" dentistry originated with doctors is true not only of America, the home of modern dentistry. Dr. Walkhoff will remember that the founder and the first president of the Central Union of German Dentists was one Prof. Heider, of whom it is said that he was the first German dentist to master the art of inserting permanent gold fillings.

Dr. Walkhoff freely admits the indebtedness of dentistry to histological research. In claiming that antiseptic treatment was applied in dentistry earlier than was the case in general medicine he appears to ignore the fact that the principles underlying this treatment did not arise within the confines of dental activities.

All along the line it may be observed that the advances in the science of dentistry—mechanics excepted—have come not from "dentists" but from dental surgeons, who are equipped with the medical degree.—From the *Oesterreichische Zeitschrift fur Stomatologie*—much condensed.—*Dental Record.*

